# Characterization of Water States in Canola Seeds With Varying Moisture Contents Using Differential Scanning Calorimetry

**DOI:** 10.1111/1750-3841.70698

**Published:** 2025-11-17

**Authors:** Vijay Balaji Kalyanakumar, Fuji Jian, Trust Beta

**Affiliations:** ^1^ Department of Biosystems Engineering University of Manitoba Winnipeg Manitoba Canada; ^2^ Department of Food and Human Nutritional Sciences University of Manitoba Winnipeg Manitoba Canada

## Abstract

Characterization of water status in grains is essential for grain drying, storage, and handling. A state diagram of canola seeds was developed incorporating the freezing curve, glass transition line, and critical moisture content (MC) at which freezable water first appears using differential scanning calorimetry. Cooling and heating rates of 1, 2, 5, 10, and 20°C/min were tested, and 1°C/min was used to determine the freezing and melting parameters such as peak temperature, onset temperature, and enthalpy. Below 17.8% MCs, the freezing point varied, and the minimum was about −26°C, whereas, beyond 17.8% MC, the freezing point increased to −6.72 ± 0.18°C at 35.8% MC. The critical MC was about 16%. Below this threshold, all remaining water exists as unfreezable water. Multiple glass transitions were observed across all tested MCs, with the first glass transition becoming undetectable above 6.2% MCs. As MC increased, the temperatures of the second and third glass transitions decreased, reaching 63.46 ± 0.39°C and 73.38 ± 0.98°C, respectively, at 35.8% MC. The developed state diagram will be useful for understanding effects of MC and temperature on the water state, and it will offer guidance for drying, handling, and storage strategies for canola seeds.

**Practical Applications:**

The state diagram aims to elucidate changes in water state as a function of MC and temperature, ultimately informing the selection of suitable conditions for handling and storage of canola. In addition, the state diagram will be used to determine the material state and knowledge about the material state is necessary for selecting suitable drying conditions.

## 1 Introduction

Water status significantly affects the physical, chemical, and biological properties of the crop seeds (referred to as grain hereafter). Water status is characterized by its moisture content, water distribution, and state, including bound, semi‐bound, free, immobile, mobile, freezable, unfreezable, absorbed, and adsorbed water. The water state in grain is influenced by moisture content (MC) and temperature. For example, when harvested grain has an MC higher than the recommended safe storage MC, the grain must be dried. The safe storage MC of canola (*Brassica napus*) is 8% (Sun et al. 2014). The drying rate is influenced by the grain's moisture content. During grain storage, quality loss such as reduced germination is largely attributed to mold growth, and moisture content is a key factor driving mold proliferation (Jian and Jayas 2022). Grain moisture status influences its physical and chemical properties, including bulk density, porosity, repose angle, specific heat, thermal conductivity, and safe storage time. Environmental factors such as temperature and relative humidity (RH) significantly affect moisture (water) dynamics during grain drying and storage. Therefore, understanding water status (moisture content) is crucial for designing equipment, optimizing operational parameters, and developing effective management practices for harvested, stored, and handled grain.

At low to intermediate grain moisture content (water activity <0.7), bound and semi‐bound water are closely associated with biomaterials, reducing water availability. Bound water is defined as monolayer water molecules bonded to biomaterials via hydrogen bonds, whereas semi‐bound water consists of multilayer water molecules. At sufficiently high water activity (>0.7), the free water content increases and exhibits properties similar to bulk water. Nuclear magnetic resonance can be used to measure bound, semi‐bound, and free water content (Wang et al. 2024). Sorption isotherm studies fundamentally characterize bound, semi‐bound, and free water at temperatures above 0°C (Zomorodian et al. 2011). Recently, Tiwari and Jian (2025) extended this approach to sub‐zero temperatures (<0°C) for canola. They observed a Type II isotherm curve for canola stored at −25°C, demonstrating the existence of free water even at these sub‐zero conditions. At sub‐zero temperatures, free water forms ice and is therefore known as freezable water. This transformation can cause cell damage and potentially lead to seed death. In contrast, bound and semi‐bound water remains as unfreezable water (Sun et al. 2023). Germination tests on stored peas, soybeans, and canola with different MCs at sub‐zero temperatures have been used to determine the critical MC and temperature thresholds beyond which germination rapidly declines (Tiwari and Jian 2025; Vertucci 1989). The decline in germination is attributed to ice formation; however, studies indicate that seed viability at sub‐zero temperatures is also influenced by biochemical changes associated with storage MC, temperature, and time (Jaganathan et al. 2017; Li et al. 2022; Roberts and Ellis 1989). While Gusta et al. (2006) offer insights on seed viability of imbibed canola seeds, the critical MC and temperature at which freezable water forms ice are not well understood.

A state diagram is a graphical representation of temperature (y‐axis) versus MC (x‐axis), illustrating various state transitions, such as freezing and glass transition as a function of MC. State diagrams are used to determine the unfreezable and freezable water state, freezing point, and glass transition temperature. For the grain and food industry, they are widely employed to understand how material state changes with temperature and MC and to determine suitable processing temperatures and explain the biochemical changes during food storage (Rahman 2006). Although various techniques are available to develop state diagrams, differential scanning calorimetry (DSC) is commonly used in determining the freezing point and glass transition temperature of food materials due to its ease of sample preparation and straightforward procedure (Rahman 2009). DSC has been used to develop state diagrams for various food materials, including fruits and vegetables, meat, poultry, and fish. State diagrams have been developed for crop seeds such as rough rice (Ameyaw Owusu et al. 2024; Sablani et al. 2009), corn (Williams and Leopold 1989), soybean (Bruni and Leopold 1991), and beans (Chigwedere et al. 2019; Kyomugasho et al. 2021). However, there is no state diagram available for canola.

Therefore, the primary objective of this study is to determine the critical MC at which freezable water appears and to develop a state diagram with freezing and glass transition curves for canola seeds using DSC. The state diagram aims to elucidate changes in water state as a function of MC and temperature, ultimately informing the selection of suitable conditions for seed drying, handling, and storage of canola.

## 2 Materials and Methods

### 2.1 Canola and Canola Oil Preparation

Canola seeds (hereafter referred to as canola) (LP223 variety) were purchased from a farmer in Brandon, Manitoba, Canada. Dockage was removed from the purchased canola using a sieve shaker (AS 400, Retsch GmbH, Haan, NRW). Canola was then collected from mesh #10, 12, and 16 with openings of 2.0, 1.70, and 1.18 mm, respectively. To assess the quality of the purchased seeds, moisture content and germination were determined, ensuring the tested seeds represented a viable population. The MC of canola (wet basis, unless otherwise specified) in triplicates by drying 10 g samples at 130°C for 4 h, following ASAE Standard S352.2 (ASABE 2017). The MC of the purchased canola was 8.4% ± 0.1%. The germination of canola was determined by following the procedure developed by Wallace and Sinha (1962). Twenty‐five whole seeds were placed on Whatman #3 paper in a Petri dish containing 5 mL of distilled water. The dishes in triplicates were maintained at 21°C under room light conditions for 7 days. The germination was 96% ± 0.65%, indicating a good quality of the canola used in this study. The purchased canola was dried from 8.4% to 3% MC by spreading it on the floor in an environmental room maintained at 25°C and 27% RH for eight weeks. To produce canola with 4% to 36% MCs, water was directly added to the canola following the procedure used by Jian et al. (2012). For example, to create 4% MC, about 32 mL of distilled water was added to 3 kg of 3% MC canola, and the mixture was tumbled for 15 min. After 24 h, the canola was mixed again for 15 min. The tempered canola was transferred to a double plastic bag, sealed with rubber bands, and stored for 14 days in an environmental chamber (CMP 4030, Controlled Environments Limited, Winnipeg, MB) set at 5°C and 80% RH. This storage period and environment ensured the seed fully absorbed the added water.

To investigate whether the oil in the seed is related to the thermal transition of canola seed, canola oil was extracted and tested. The extraction was performed using a Goldfisch fat extractor (Labconco, Kansas City, MO). About 50 g of canola was first dried at 130°C for 4 h, then ground for approximately 5 min to a fine powder using a laboratory grinder (Seedburo, Des Plaines, IL). The particle size of the ground sample was not determined. A sample packet was prepared by enclosing 5 g of the ground powder within filter paper #5. One sample packet was placed in each sample tube, which was placed inside the beaker. Petroleum ether, 25 mL–30 mL, was poured into the beaker. The beaker was placed over the heater and tightened to the fat extractor. After 16 h, the sample tube was removed from the beaker. The beaker was placed in the hot air oven at 130°C to evaporate petroleum ether. The extracted oil was transferred to a separate beaker, sealed, and refrigerated until further testing.

### 2.2 DSC Thermal Analysis

#### 2.2.1 Sample and Reference Pan Preparation

All thermal analyses were carried out using a Discovery DSC 250 (TA Instruments‐Waters, New Castle, DE) equipped with a mechanical refrigerator (RCS 90). Nitrogen was used as both the cooling gas and purge gas, with a flow rate of 50 mL/min. A single whole seed (4 mg to 6 mg) of the prepared canola was placed in an aluminum pan (40 µL volume), which was sealed to create the sample pan. A reference pan was prepared using the same pan and procedure, but without canola.

#### 2.2.2 DSC Procedure

The cool‐hold‐heat method was employed, involving cooling the sample from 20 or 40°C to –90 or 0°C, holding isothermally for 5 min, and then heating to 20°C or 120°C. Various cooling and heating rates were applied to measure different parameters, as outlined in Table 1. To optimize the rates, cooling and heating rates of 1, 2, 5, 10, and 20°C/min were tested, with an optimized rate of 1°C/min was selected for determining the freezing and melting characteristics of the prepared canola and oil. A heating rate of 10°C/min was used to determine the glass transition temperature.

**TABLE 1:** DSC procedure, sample analysis at various MCs, and parameters determined.

Testing	Sample (MC, %) ^a^	DSC procedure ^b^	Parameters
Optimum cooling rate	Canola (3.8)	Cooling from 20 to –90°C at 1, 2, 5, 10, and 20°C/min, heating from –90 to 20°C at 10°C/min	Enthalpy of freezing and melting
Optimum heating rate	Canola (3.8)	Cooling from 20 to −90°C at 2°C/min, heating from –90 to 20°C at 1, 2, 5, 10, and 20°C/min	Enthalpy of freezing and melting
Freezing and melting characteristics	Canola (3 to 36) Canola oil	Cooling from 20 to –90°C at 1°C/min, heating from –90 to 20°Cat 1°C/min	Enthalpy, onset temperature, and peak temperature of freezing and melting
Glass transition	Canola (3 to 36)	Cooling from 40 to 0°C at 5°C/min, heating from 0 to 120°C at 10°C/min	Glass transition temperature

^a^ Canola = whole single canola seed, MC = Moisture content (wb, %).

^b^ Isothermally held for 5 min at ‐90 or 0°C before heating.

#### 2.2.3 Thermogram Analysis

**FIGURE 1:**
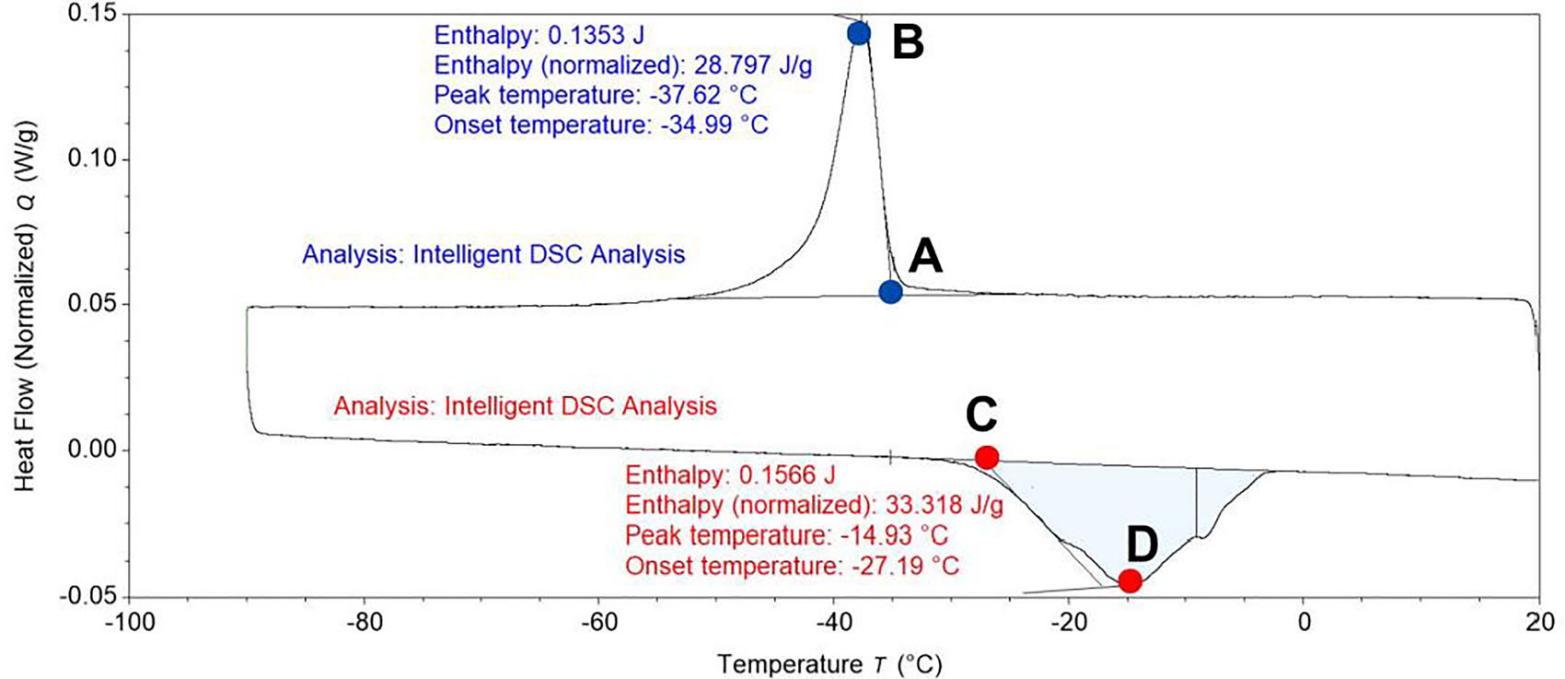
Thermogram analysis of 3.4 % MC canola cooled from 20 to −90°C at 1°C/min, held at −90°C for 5 min, and heated from −90 to 20°C at 1°C/min. A and C are onset temperatures (the intersection of the baseline and tangent of the inflection point); B and D are peak temperatures (the temperature at which maximum heat flow is recorded); enthalpy is the area under the exothermic and endothermic peaks.

**FIGURE 2:**
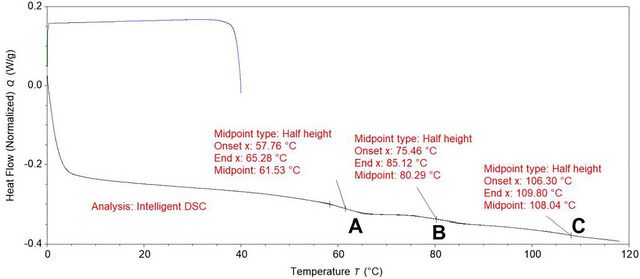
Thermogram analysis of 3.4% MC canola cooled from 40 to 0°C at 5°C/min, held at 0°C for 5 min, and heated from 0 to 120°C at 10°C/min. A, B, and C are glass transitions characterized by the shift in the thermogram.

A thermogram with temperature on the x‐axis and normalized differential heat flow (W/g) on the y‐axis was obtained (Figures 1 and 2). The exothermic peak, endothermic peak, and shift in the thermogram were analyzed using TRIOS software (version 5.2; TA Instruments‐Waters, New Castle, DE). The TRIOS Intelligent function was used to calculate the enthalpy, onset temperature, and peak temperature of the peaks, as well as the onset, midpoint, and endpoint of the glass transition (Figures 1 and 2). Enthalpy was determined as the area under the exothermic peak with a linear baseline (for canola with less than 15.9% MC and canola oil) or sigmoidal baseline (for canola with more than 17.8% MC). The onset temperature was defined as the intersection of the baseline and the tangent of the inflection point (Figure 1). The peak temperature was the temperature at which maximum heat flow was recorded (Figure 1). Glass transition was identified as the shift in the thermogram, determined using the software analysis settings (Figure 2). The midpoint temperature was reported as the glass transition temperature (*T*_g_).

### 2.3 Calculation of Unfreezable and Freezable Water Content

**FIGURE 3:**
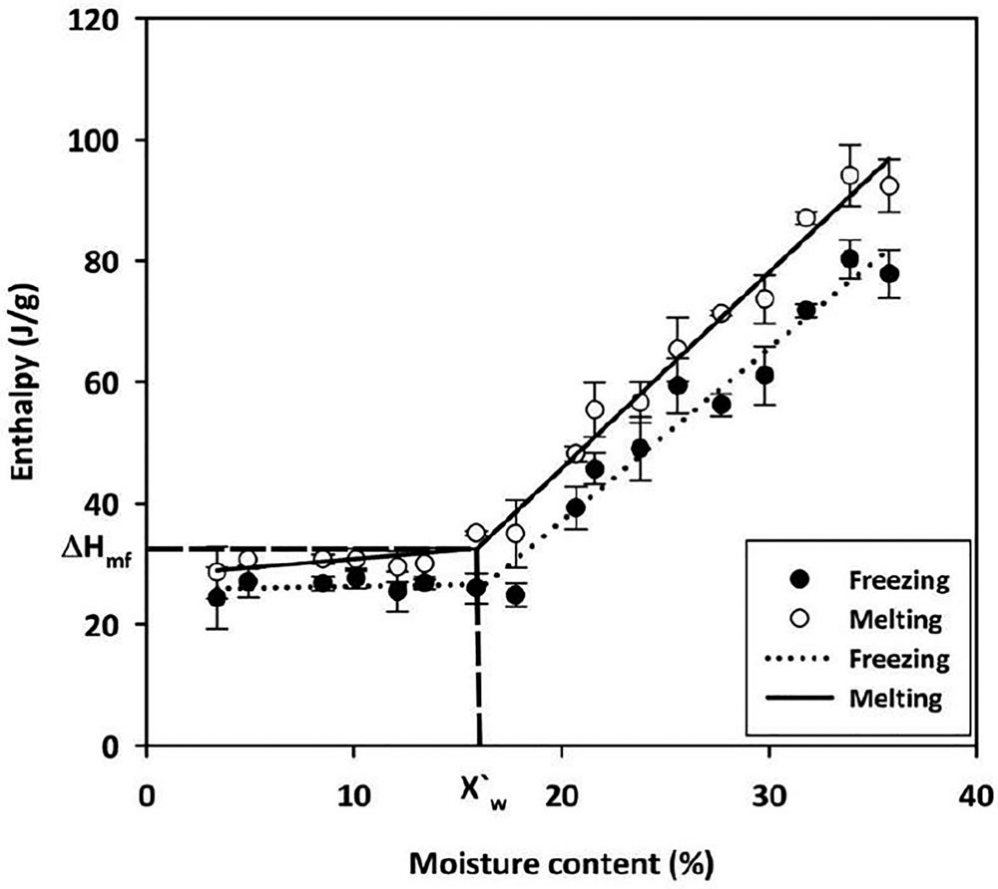
Enthalpy of freezing and melting of canola with various MCs (3.4 to 35.8%). In the graph, *X*`_w_ = 16.0 % is the unfreezable water content and Δ*H*_mf_ = 33.61 J/g is the fat melting enthalpy. Lines represent predicted values. For MCs 27.7 to 35.8%, where two separate peaks were observed, freezing enthalpy is the sum of enthalpies of the two peaks.

The critical MC at which freezable water first appears was determined from the melting enthalpy and canola MCs, as described by Vertucci (1989). Below this critical MC, freezable water is either absent or present in negligible amounts, undetectable by the DSC. Above this critical MC, the freezable water forms ice and is detectable by the DSC. The melting enthalpy was determined using TRIOS intelligent analysis software. These resulting enthalpies at different canola MCs are presented in Figure 3. A piecewise linear model was regressed for the melting enthalpy against MC, resulting in two segments (Figure 3). The point of intersection (*X*′_w_) of the two segments represents the critical MC at which freezable water first appears (Figure 3) (Vertucci 1989).

The mass of freezable water present in the canola is normally calculated from the ice melting enthalpy (Ross 1978). The ice melting enthalpy is the difference between the melting enthalpy detected by the DSC and the fat melting enthalpy, where the latter was calculated based on Figure 3. The mass of freezable water was determined as the ratio of the ice melting enthalpy of the sample to the enthalpy of pure water at 0°C (Δ*H*_o_ = 334.66 J/g). The mass of unfreezable water was calculated as the difference between the total water mass and the mass of freezable water (Roos and Karel 1991):

(1) $$W_{FW}=\frac{\Delta H_{ice}}{\Delta H_{o}}$$

(2) $$W_{UFW}=W_{TW}-W_{FW}$$

where *W_FW_* is the mass of freezable water (g), *W_UFW_* is the mass of unfreezable water (g), *W_TW_* is the total water mass (g), and *ΔH_ice_* is the ice melting enthalpy (J). The unfreezable and freezable water content was calculated using the method described by Li et al. (1998). The unfreezable water content on a wet sample mass basis equals the ratio of the unfreezable water mass to the wet sample mass (Equation 3). The unfreezable water content on a dry sample mass basis equals the ratio of the unfreezable water mass to dry sample mass, calculated as the wet sample mass minus the total water mass (Equation 4). The freezable water content on a wet sample mass basis equals the ratio of the freezable water mass to the wet sample mass (Equation 5). The freezable water content on a dry sample mass basis equals the ratio of the freezable water mass to the dry sample mass, calculated as the wet sample mass minus the total water mass (Equation 6):

(3) $$MC_{UFW}\left(wet\;sample\;basis\right)=\frac{W_{UFW}}{W_{S}}\cdot 100$$

(4) $$MC_{UFW}\left(dry\;sample\;basis\right)=\frac{W_{UFW}}{W_{S}-W_{TW}}\cdot 100$$

(5) $$MC_{FW}\left(wet\;sample\;basis\right)=\frac{W_{FW}}{W_{S}}\cdot 100$$

(6) $$MC_{FW}\left(dry\;sample\;basis\right)=\frac{W_{FW}}{W_{S}-W_{TW}}\cdot 100$$

where *W_UFW_* is the unfreezable water mass (g), *W_FW_* is the freezable water mass (g), *W_TW_* is the total water mass (g), and *W_S_* is the sample mass (g).

### 2.4 Data Analysis

All experiments were conducted in triplicate, and the mean with SD of three values is reported. Statistical analysis was performed using SigmaPlot 11.0 (Grafiti LLC., Palo Alto, USA). The Pearson's correlation coefficient (r) was determined between enthalpy (freezing and melting) and cooling rates, as well as between enthalpy (freezing and melting) and heating rates. Non‐linear regression models were fitted to the freezing enthalpy, melting enthalpy, and freezing point at various moisture contents. A simple linear regression model was fitted to the glass transition temperature against MC. The goodness of fit was assessed using global and segmented correlation coefficients (R) and root mean square errors (RMSE) for linear models. RMSE was used for nonlinear models.

## 3 Results

### 3.1 Relationship Between Enthalpy and Cooling or Heating Rate

The freezing enthalpy of 3.8% MC canola was 28.62 ± 1.45, 27.79 ± 2.09, 23.44 ± 3.82, and 13.58 ± 0.22 J/g at the cooling rates of 1, 2, 5, and 10°C/min, respectively. No freezing enthalpy was detected at 20°C/min. The melting enthalpy was 32.74 ± 0.73, 30.14 ± 2.21, 28.24 ± 4.62, 28.86 ± 2.38, and 25.17 ± 3.38 J/g at cooling rates of 1, 2, 5, 10, and 20°C/min, respectively. Both freezing and melting enthalpy decreased with increasing cooling rate. There was a significant correlation between the freezing enthalpy and cooling rate (*r* = −0.99, *p* < 0.01) and between melting enthalpy and cooling rate (*r* = −0.90, *p* = 0.04). Thus, the cooling rate affected freezing and melting enthalpy, with lower cooling rates resulting in higher freezing and melting enthalpy. Higher freezing and melting enthalpy would generate a higher accuracy of the determined freezing and melting parameters, such as freezing point. Therefore, a cooling rate of 1°C/min was used in this study.

The freezing enthalpy of 3.8% MC canola was 26.90 ± 3.41, 29.20 ± 0.84, 23.88 ± 5.17, 27.78 ± 2.09, and 25.96 ± 0.14 J/g at heating rates of 1, 2, 5, 10, and 20°C/min, respectively. The melting enthalpy was 30.19 ± 2.07, 31.02 ± 0.95, 26.60 ± 3.56, 30.14 ± 2.21, and 29.44 ± 2.64 J/g at the corresponding heating rates. There was no significant correlation between the freezing enthalpy and heating rate (*r* = −0.23, *p* = 0.71) or between melting enthalpy and heating rate (*r* = −0.09, *p* = 0.87). Thus, the heating rate did not affect freezing and melting enthalpy. However, at the heating rate of 1°C/min, the melting thermogram showed a broad transition with multiple peaks. Therefore, a heating rate of 1°C/min was selected for this study, as it provided the detailed information necessary from the broad transition.

### 3.2 Freezing and Melting Characteristics

**FIGURE 4:**
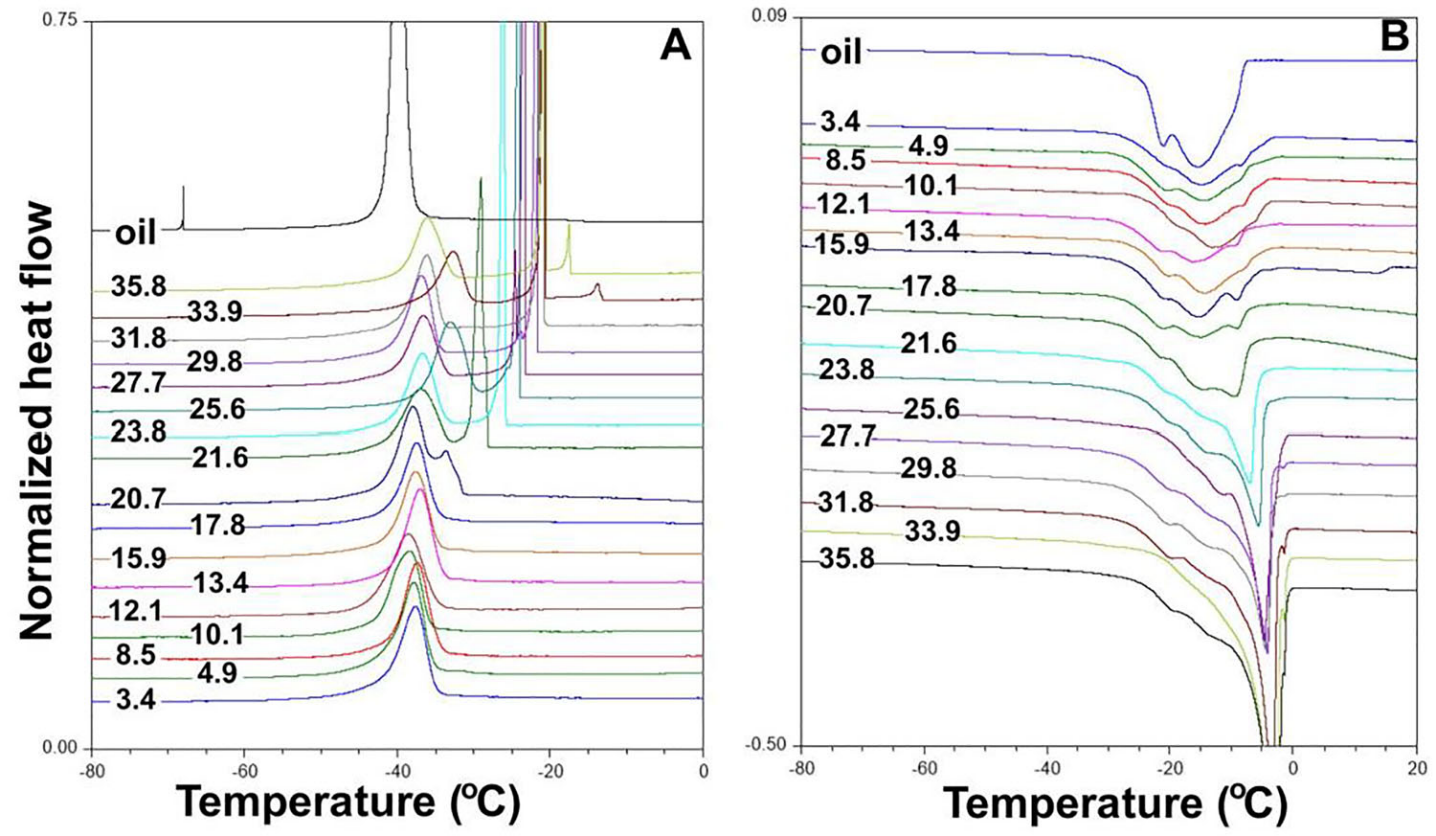
Freezing (A) and melting (B) thermograms of canola oil and canola seed with various MCs (3.4 to 35.8%). In the graph, the number on each line is the moisture content of the canola seeds (%). “Oil” is the canola oil. The normalized heat flow is not the actual values. Baselines of each thermogram were adjusted to prevent overlapping of the peak.

The freezing and melting thermograms of canola oil are presented in Figure 4. During freezing, a single narrow peak was observed with an onset temperature of −38.44°C ± 0.03°C and a peak temperature of −39.80°C ± 0.04°C. In contrast, during melting, broad multiple peaks, ranging from −37°C to ‐6°C, were observed with an onset temperature of −24.74°C ± 0.08°C and peak temperature of −15.33°C ± 0.16°C (Teles dos Santos et al. 2016).

The freezing thermograms of canola with MCs ranging from 3.4% to 17.8% exhibited a single broad peak with one onset temperature and one peak temperature (Figure 4A). At 3.4% MC, the peak was broader than that of canola oil, with a peak temperature of −36.68°C ± 1.00°C, which was close to the peak temperature of canola oil. The low water content increased the peak temperature of oil freezing in the seed by about 2°C, possibly due to bound and absorbed water (Zomorodian et al. 2011). As the MC increased from 3.4% to 17.8 %, the peak temperature showed a slight increase (the peak temperature at 17.8 % MC was −38.42°C ± 1.35°C), and the peaks resembled those of canola oil. Therefore, the broad peak was primarily attributed to oil freezing in the seed at ≤17.8% MC. Water in these MCs likely comprises bound, absorbed, adsorbed, and free water (Dong et al. 2025). For canola with 20.7% to 25.6% MC, however, two peaks were observed. One of the two observed peaks was similar to the oil freezing peak seen at ≤17.8 % MC, while the other peak occurred at a higher temperature, potentially caused by free water. As the MC further increased, the water freezing peak (associated with free water) gradually became a narrower and shifted to a higher temperature. Notably, the oil freezing peak remained unaffected throughout this MC range. The water freezing peak temperatures for canola with 20.7, 21.6, 23.8, 25.6, 27.7, 29.8, 31.8, 33.9, and 35.8% MC were observed to be −34.63, −29.34, −26.25, −24.73, −23.22, −21.08, −20.71, −19.96, and −19.20°C, respectively. The water freezing peak was observable only when the canola MC was ≥17.8%. Crucially, higher MCs resulted in higher peak (freezing) temperatures, and the peak was not observed when the canola MC was less than 17.8%.

The freezing enthalpy of 3.4% MC canola was 24.36 ± 5.07 J/g. As the MC increased to 17.8 %, the freezing enthalpy showed minimal variation (freezing enthalpy of 17.8 % MC was 24.86 ± 1.96 J/g). Therefore, the freezing enthalpy was primarily attributed to oil freezing in canola with ≤17.8% MC. Beyond 17.8% MC, the freezing enthalpy of canola drastically increased from 24.86 ± 1.93 J/g at 17.8% MC to 77.80 ± 4.03 J/g at 35.8% MC (Figure 3). This increase in enthalpy was likely due to the increased amount of free water when the canola MC was higher than 17.8 %. Assuming the freezing enthalpy of the oil inside the seed was 24.4 J/g, the enthalpy used by water in canola with 20.7, 21.6, 23.8, 25.6, 27.7, 29.8, 31.8, 33.9, and 35.8 % MC canola would be 14.8, 21.3, 24.6, 34.6, 39.1, 42.9, 57.5, 65.4, and 61.9 J/g, respectively, calculated by subtracting the oil's enthalpy contribution from the total freezing enthalpy.

**FIGURE 5:**
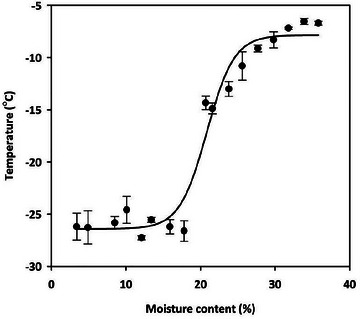
Freezing point of canola with various MCs (3.4 to 35.8%). Line represents predicted values.

The melting thermograms of canola with different MCs are shown in Figure 4B. Multiple peaks with one onset temperature and one peak temperature were observed throughout the MC range of 3.4% to 35.8%. The onset temperature (*T_f_*) was considered the freezing point. At 3.4% MC, the onset temperature and peak temperature were −26.20 ± 1.29 and −13.65°C ± 1.71°C, respectively, which was close to the melting onset and peak temperatures of canola oil. As the MC increased to 17.8%, the onset and peak temperatures showed minimal variation. At this MC, the onset temperature was –26.62 ± 0.99, and the peak temperature was ‐13.42°C ± 2.18°C, with the peaks resembling those of canola oil melting. However, the peaks gradually changed, becoming wider and shallower, indicating that the *T_f_* of the peak became higher than that of the oil. At 15.9% MC, a small peak appeared at a higher temperature than the canola oil melting peak, suggesting it was caused by ice melting. Therefore, the endothermic peak up to 15.9% MCs was attributed to oil melting, while the endothermic peak from 15.9% to 17.8% MC was due to oil and ice melting. As the MC increased from 17.8% to 35.8%, the ice melting peak at the higher temperature gradually became narrower and deeper. Simultaneously, the freezing point (*T_f_)* drastically increased, reaching −6.72°C ± 0.18°C at 35.8% MC. Thus, water content ≥17.8% MC significantly influenced the freezing point of canola. A sigmoidal model was fitted to *T_f_* and MCs, as *T_f_* increased non‐linearly with MC (Figure 5).

The melting enthalpy was 28.57 ± 4.32 J/g at 3.4% MC. It gradually increased to 34.95 ± 5.60 J/g up to 17.8% MC, and beyond this point, the melting enthalpy dramatically increased to 92.32 ± 0.89 J/g at 35.8% MC. This increase in melting enthalpy was attributed to the increase in water content, as the oil content in the canola remained constant in this study. Both freezing and melting enthalpy changed non‐linearly with MC (Figure 3). To model this relationship, 2‐segment piecewise linear models were fitted to freezing enthalpy and melting enthalpy at the measured MCs. In both models, the point of intersection of the two segments was approximately 16% MC (Figure 3). This suggests that freezable water would first appear around 16% MC, and water content in the seed at <16% MCs could exist as unfreezable water.

### 3.3 Freezable and Unfreezable Water Content

**FIGURE 6:**
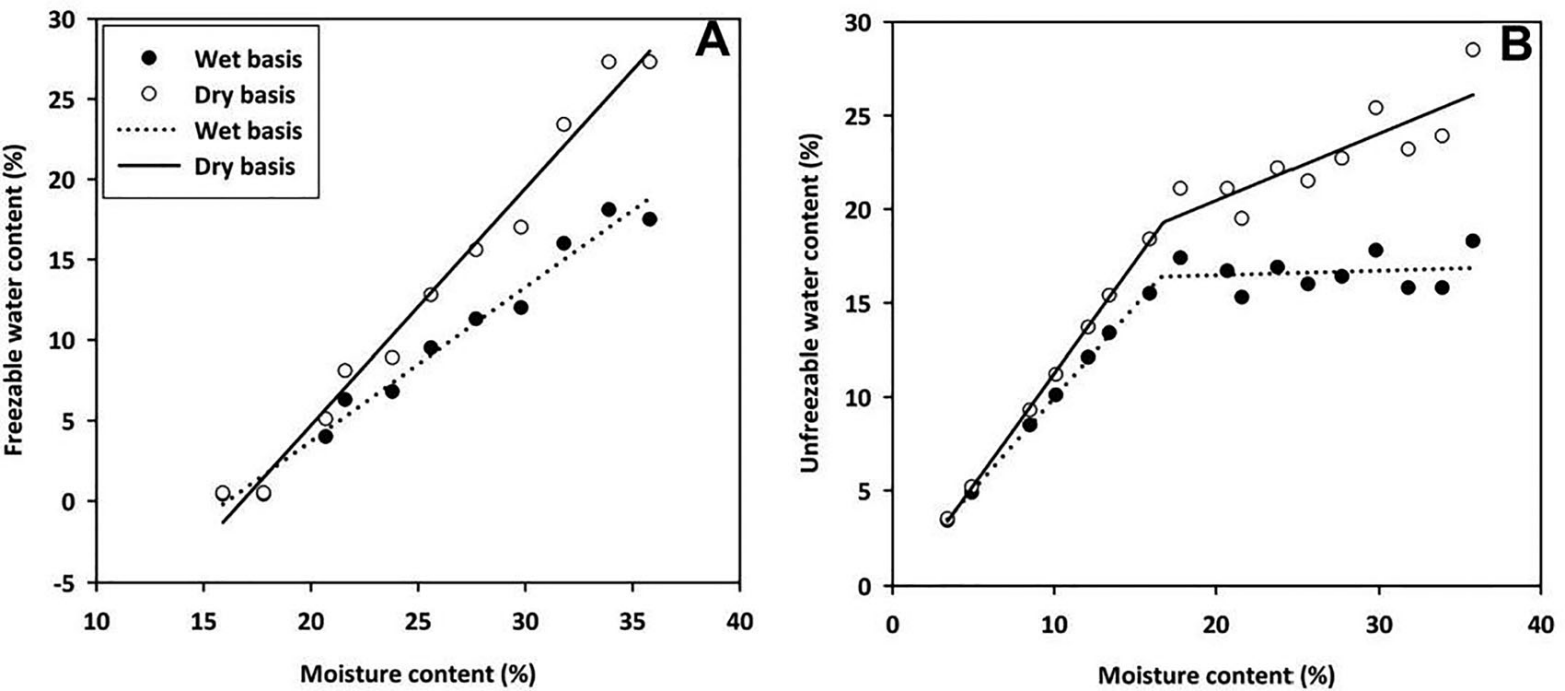
Freezable water content (A) and unfreezable water content (B) of canola at various MCs (3.4 to 35.8%). The symbols represent the calculated values and lines represent the values predicted by the developed regression equations.

The mass of freezable water in canola from 3.4% to 15.9% MC could not be determined due to the ice melting enthalpy being 0 J/g in this study. Therefore, freezable water content was only determined for canola MCs ranging from 15.9% to 35.8%. For instance, at 15.9% MC with a canola seed mass of 5.1 mg, the mass of freezable water was 0.022 mg based on the ice melting enthalpy. This resulted in a calculated freezable water content on a wet sample mass basis of 0.4%. At 35.8% MC, the calculated freezable water content on a wet sample mass basis was 17.5%. The freezable water content in the seed above 15.9% MCs increased linearly with the increase in MC (Figure 6). Unfreezable water content was modeled using a 2‐segment piecewise linear model (Figure 6), consistent with the observation that freezable water would first appear around 16% MC, and water content in the seed at <16% MC could exist as unfreezable water.

### 3.4 Glass Transition Characteristics

**FIGURE 7:**
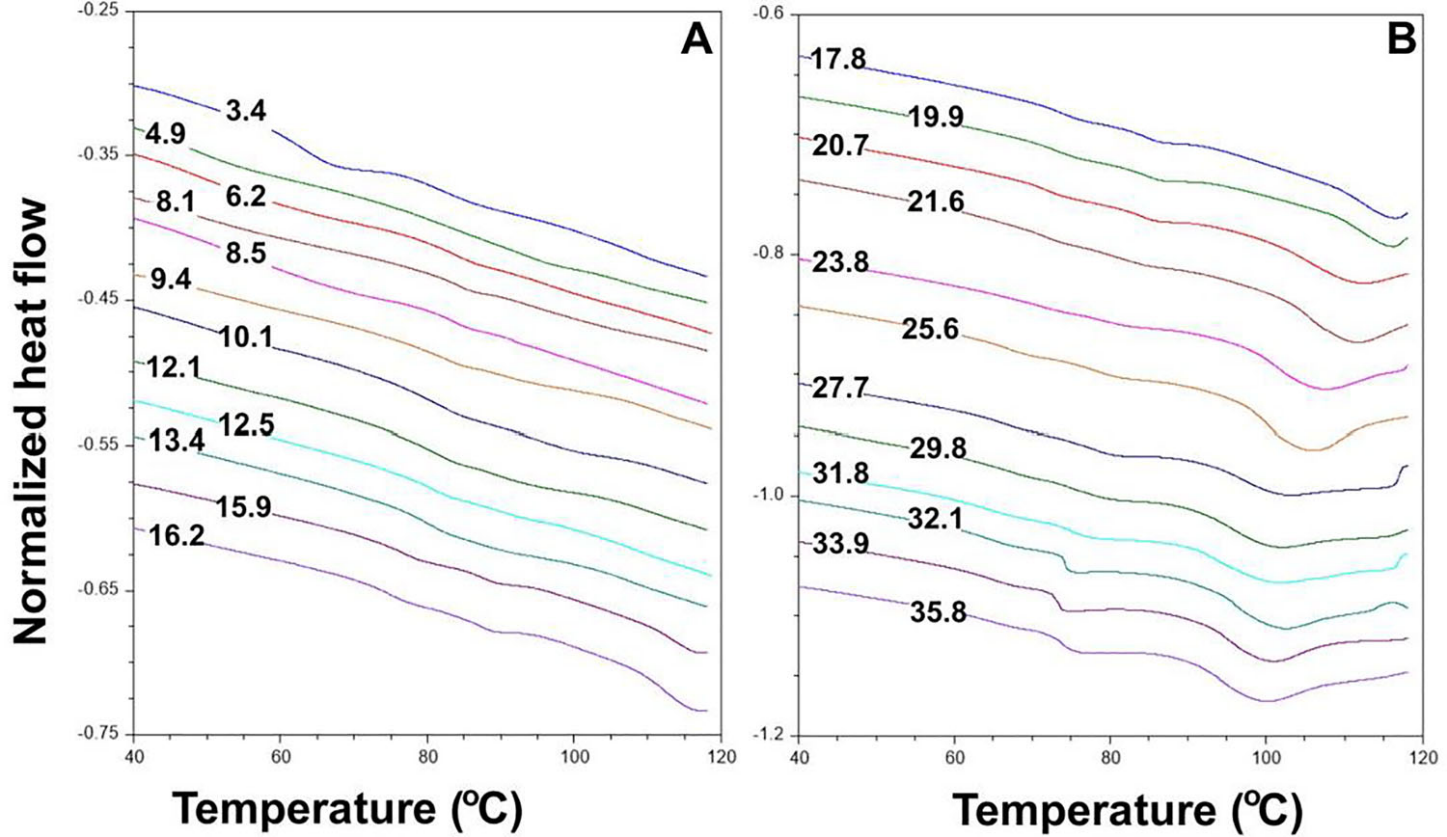
Thermograms of canola at various MCs (A) 3.4 to 16.2% and (B) 17.8 to 35.8%. The normalized heat flow is not the actual value. Baselines of each of the thermograms were adjusted to prevent overlapping.

Multiple glass transitions were observed (Figure 7). The glass transitions were ranked by their onset temperatures (from lowest to highest, corresponding to the order they appeared during the test): glass transition‐one (*T_g1_*), glass transition‐two (*T_g2_*), and glass transition‐three (*T_g3_*). For moisture contents ranging from 3.4 to 6.2%, three glass transitions (*T_g1,_ T_g2,_* and *T_g3_*) were observed. In contrast, for MCs between 8.1% and 35.8%, two glass transitions (*T_g2_* and *T_g3_*) were observed. The onset temperatures for these transitions ranged from 46.83 to 58.65, 60.58 to 81.14, and 72.70 to 94.54°C for *T_g1,_ T_g2_*_,_ and *T_g3_*, respectively. Given that canola oil remains liquid at temperatures above −25°C, the observed glass transitions were likely caused by the status of water and chemical compounds other than oil in the canola.

**FIGURE 8:**
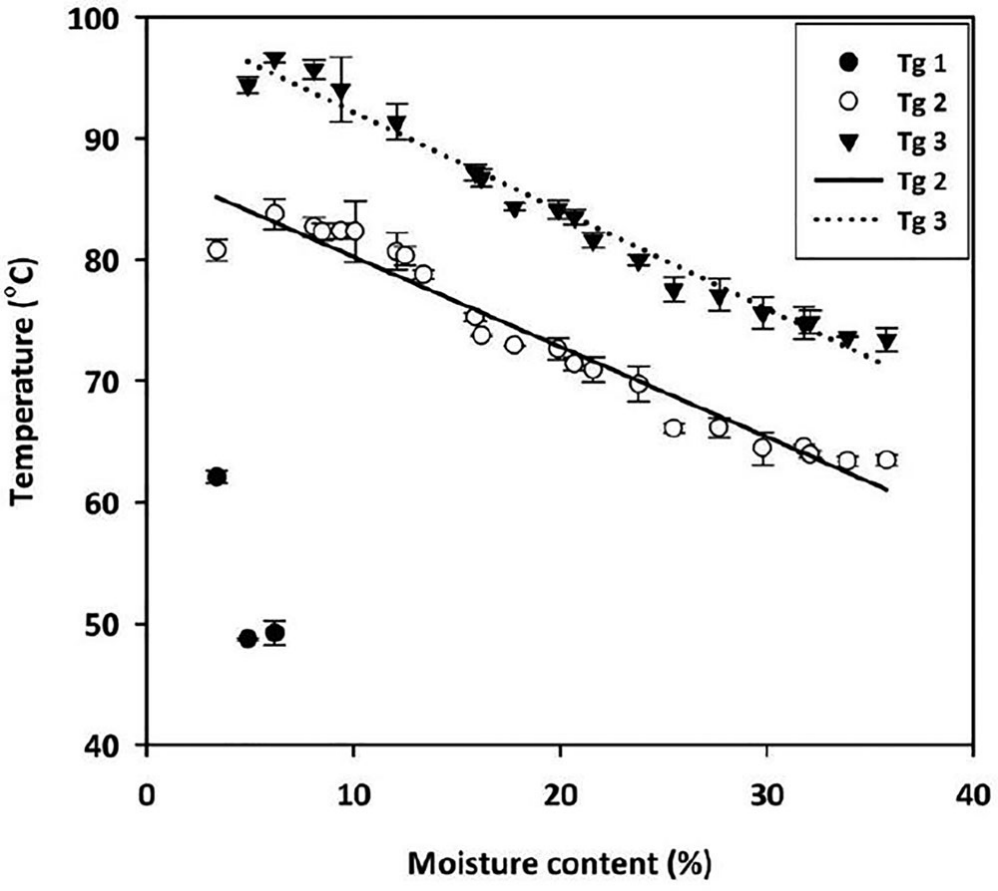
Glass transition temperature of canola at various MCs (3.4 to 35.8%). In the legends, *T*_g1_, *T*_g2_, and *T*_g3_ represent glass transition‐one, ‐two, and ‐three, respectively. Lines represent predicted values.

The glass transition temperatures (*T_g_*, midpoint temperature) for *T_g1_* and *T_g2_* at 3.4% MC were 62.08 ± 0.52 and 80.79 ± 0.87°C, respectively, while *T_g3_* was not detected. At 6.2% MC, the *T_g_* values were 49.23 ± 1.02, 83.76 ± 1.25, and 96.65 ± 0.40°C, respectively, for *T_g1_, T_g2_*, and *T_g3_*. For MCs of 8.1% and above, only *T_g2_* and *T_g3_* were observed, with values of 82.69 ± 0.82 and 95.67 ± 0.40°C, respectively, at 8.1% MC. As the MC increased to 35.8%, *T_g2_* and *T_g3_* decreased to 63.46 ± 0.39 and 73.38 ± 0.98°C, respectively. The *T_g_* values decreased linearly with increasing MC (Figure 8). There was a significant correlation between *T_g2_* and *T_g3_* at the determined MCs, but no significant correlation was found between *T_g1_* and MC. Therefore, linear models were fitted only for *T_g2_* and *T_g3_* values (Table 2).

**TABLE 2:** Regression equations to predict the properties of canola at various moisture contents.

			Coefficients				
Properties	MC (%, wet basis)	r	a	b	c	d	RMSE
ΔH_f_	3.4 – 35.8^a^	‐	25.77	5.34	284.60	0.16	3.06
	3.4 – 16.4^b^	0.21^d^	25.78	5.25	‐	‐	1.01
	16.4 – 35.8^b^	0.97^e^	−19.89	284.53	‐	‐	3.86
ΔH_m_	3.4 – 35.8^a^	‐	27.95	28.78	323.51	0.16	3.76
	3.4 – 15.9^b^	0.63^d^	27.96	28.57	‐	‐	1.48
	15.9 – 35.8^b^	0.99^e^	−17.18	317.41	‐	‐	2.99
*T*_f_	3.4 – 35.8^c^	—	−26.42	18.56	0.21	0.02	1.47
*T*_g2_	3.4 – 35.8^b^	−0.97^e^	87.69	−74.38	‐	‐	1.63
*T*_g3_	3.4 – 35.8^b^	−0.99^e^	100.31	−81.24	‐	‐	1.21
MC_UFWa_	15.9 – 35.8^b^	0.30^d^	15.39	4.44	—	—	0.89
MC_UFWb_	15.9 – 35.8^b^	0.87^e^	12.91	37.10	—	—	1.31
MC_FWa_	15.9 – 35.8^b^	0.99^e^	−15.42	95.61	—	—	0.89
MC_FWb_	15.9 – 35.8^b^	0.99^e^	−24.77	147.33	—	—	1.32

Abbreviations: MC, moisture content of canola; MC_FWa_, freezable water content on a wet sample mass basis (%); MC_FWb_, freezable water content on a dry sample mass basis (%); MCU_FWa_, unfreezable water content on a wet sample mass basis (%); MCU_FWb_, unfreezable water content on a dry sample mass basis (%); r, Pearson's correlation coefficient; RMSE, root mean square errors; *T*_f_, freezing point (°C); *T*_g2_, glass transition temperature‐two (°C); *T*_g3_, glass transition temperature‐three (°C); Δ*H_f_*, freezing enthalpy (J/g); Δ*H*_m_, melting enthalpy (J/g).

^a^ The regression equation is *Y* = a + bX + c(X−d)D, where *Y* = property in their respective units, *X* = MC in decimals, and *D* = dummy variable, If X ≤ d, *D* = 0, else *D* = 1.

^b^ The regression equation is Y = a + bX.

^c^ The regression equation is $Y=a+\frac{b}{1+exp\;(\frac{c-X}{d})}.$

^d^ There is no significant correlation (*p* > 0.05).

^e^ There is a significant correlation (*p* < 0.05).

### 3.5 State Diagram

**FIGURE 9:**
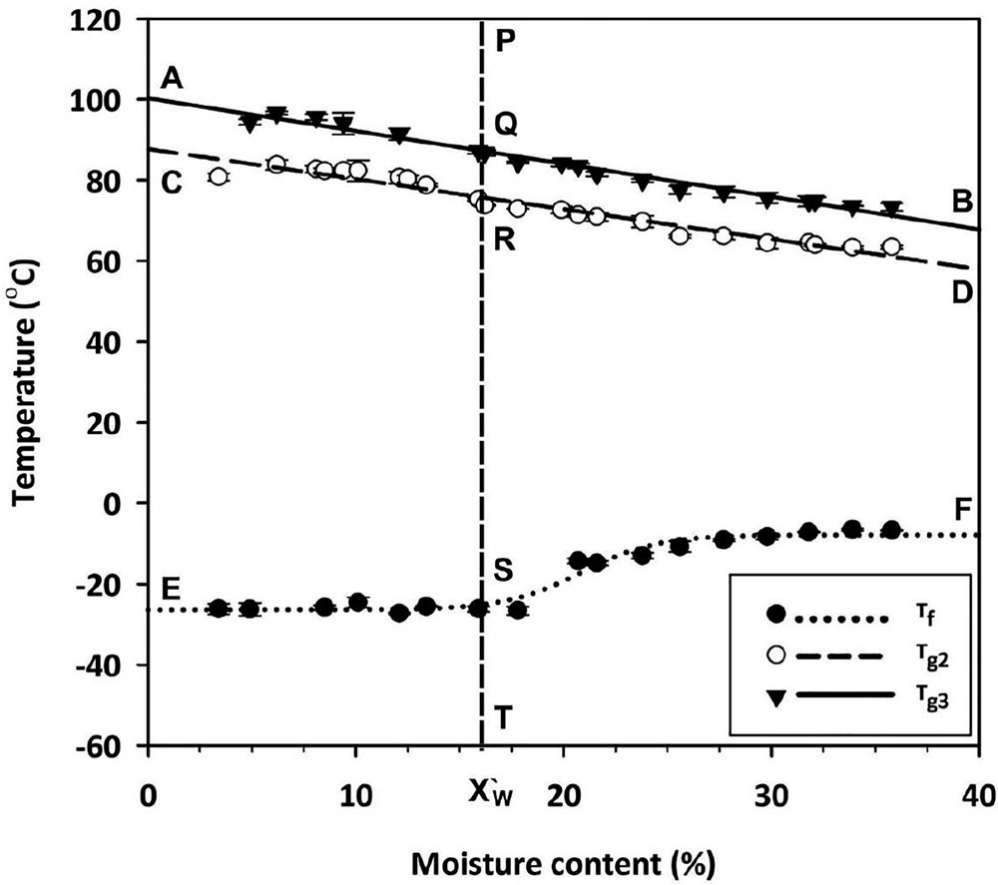
State diagram of canola seed. In the graph, *X*`_w_ = 16.0% is the unfreezable water content. In the legends, *T*_f_ represents freezing point, *T*_g2_ and *T*_g3_ represent glass transition temperatures. AQB = glass transition‐three line, CRD = glass transition‐two line, ESF = freezing curve, and PQRST = unfreezable water content line.

The state diagram of canola (Figure 9) illustrates the relationship between moisture content (MC) and temperature, featuring glass transition lines (AQB and CRD), a freezing curve (ESF), and a critical MC line (PQRST). The critical MC (*X*′_w_) was approximately 16%, marking the point where freezable water first appears. To the left of the PQRST line, all water content in the seed is unfreezable water and remains in an unfrozen state. To the right of the PQRST, both freezable and unfreezable water coexist. Below the SF line, freezable water exists as ice, while above the SF line, it exists in a liquid state. This diagram provides a comprehensive understanding of the thermal and physical state of canola as a function of MC and temperature.

## 4 Discussion

### 4.1 Canola Oil Freezing and Melting

The melting onset temperature of commercial canola oil is reported as −30°C (Teles dos Santos et al. 2016). Our observed value of −25°C may differ due to variations in oil refining processes that remove impurities. These results collectively suggest that the freezing and melting parameters of intact canola seed are influenced by the internal oil, with transitions occurring at approximately −38°C and −25°C, respectively.

### 4.2 Freezable and Unfreezable Water Content

The critical moisture content (MC), as defined by Vertucci (1989), is the limit of freezable water content in seeds—the MC at which freezable water first appears. This critical MC of canola was approximately 16.0% MC in this study. This value is lower than that in soybeans and peas, which are 18.4% and 20.4%, respectively (Vertucci 1989). Vertucci (1989) used the same method as in our study but at a 10°C/min heating rate. The different critical MCs could be due to the different chemical compositions among different crop seeds. The oil content in canola, soybean, and pea is about 40, 15, and 1.3% (Khodapanahi et al. 2012), which indicates that the hydrophilic components in canola are lower than in soybean and pea. Because canola has a higher oil content than soybeans and peas, the free water content will be greater in canola at the same MC. Since free water is freezable, canola's freezable water content is consequently higher than that of soybeans and peas at a given MC. Therefore, the critical MC of canola, as determined by DSC, should be approximately 16% or lower (Tiwari and Jian 2025).

Studies have consistently shown that ice formation can be lethal, significantly reducing the germination of seeds (e.g., canola, rice, and wheat) stored at sub‐zero temperatures (Gusta et al. 2006; Ishikawa and Sakai 1979). Tiwari and Jian (2025) found that canola stored at −15°C and 72% RH (MC of about 12%) for 25 weeks did not significantly lose germination. In contrast, the germination of canola stored at −20°C across a wider RH range (40% to 80%) was significantly reduced. To investigate this phenomenon, our group used modulated DSC to determine the critical MC, finding it to be 14% (unpublished data). This result contrasts with the 10%–12% MC range where Zomorodian et al. (2011) found free water in canola using the sorption method. Our study indicates that freezable water does not appear until the MC reaches approximately 16%. This discrepancy might be related to the differences in methodology: the DSC method may not detect small amounts of freezable water (Slade et al. 1991; Walters et al. 2010), and this undetected freezable water might form ice during the germination tests. While DSC cannot directly predict seed viability, it is a valuable tool for determining phase transition of water and oil in the seed that may ultimately influence viability. The germination test assesses seed viability, which can be affected by ice formation, irreversible biochemical change, and oil freezing when seeds are stored at sub‐zero temperatures (Crane et al. 2006; Jaganathan, Dalrymple et al. 2020; Han et al. 2017; Pirredda et al. 2023).

Based on the DSC melting thermograms (Figure 4B), oil freezing in canola occurs over a temperature range of approximately −20°C to −26°C. This temperature range is near the critical temperature of −20°C, where the germination of stored canola (MCs 8%–14%) was significantly reduced (Tiwari and Jian 2025). Therefore, it is plausible that the status of free water is affected by oil freezing. As Vertucci (1989) suggested, this interaction between oil freezing and free water likely contributes to the observed effects on seed germination, a conclusion supported by similar findings in tea seeds (Chen et al. 2012) and lime seeds (Hor et al. 2005).

In this study, the limit of freezable water content was determined from the ice melting enthalpy and MCs. However, this technique may overestimate the freezable water content because the ice melting enthalpy determined by DSC is typically higher than the actual value (Schenz et al. 1991). The ice melting enthalpy includes both the latent heat of fusion required to melt the ice and the sensible heat supplied to raise the temperature of melted water. Since DSC cannot isolate the latent heat of fusion alone, the calculated freezable water content might be slightly overestimated. Consequently, the limit of freezable water should be considered an estimated value.

Beyond 16.0% MC, the freezable water content increases due to the presence of free water, as confirmed by the freezing point and melting enthalpy measurements at different MCs. For canola with MCs ≤15.9%, the freezing point was around −26°C, but above 15.9% MC, the freezing point increased significantly with the appearance of freezable water. The heat of fusion of oil in canola seeds with <16.0% was 28.57 J/g. In contrast, for seeds with MCs >16.0%, the combined heat of fusion of oil and water was 317.41 J/g. The difference, 288.84 J/g, represents the heat of fusion of freezable water in the canola seed. Notably, this value is lower than the heat of fusion of bulk water (333.48 J/g) (Osborne 1939), suggesting that the free water in the freezable water content has some association with the chemical components present in the seed, although this association is weaker than that of bound or semi‐bound water.

### 4.3 Glass Transition Temperature

The glass transition temperature (*T*_g_) of canola decreased linearly with increasing MC below 35.8% (Figure 8). This trend is consistent with the plasticizing effect of water, where water molecules bind to hydrophilic components, occupy molecular space, and weaken intramolecular forces, thereby reducing the energy required for the glass transition (Roos 2020). The linear relationship between *T_g_* and MC in canola is similar to that observed in rough rice (Ameyaw Owusu et al. 2024; Perdon et al. 2000; Siebenmorgen et al. 2004; Sun et al. 2002). However, in other seeds like corn (Williams and Leopold 1989), soybean (Bruni and Leopold 1991), and common beans such as Canadian wonder, red kidney, and red haricot (Kyomugasho et al. 2021), *T_g_* decreases non‐linearly with increasing MC, potentially due to differences in the degree of plasticization. The discrepancy might also be attributed to the sample preparation methods used in these studies. For canola and rough rice, *T_g_* was determined using whole seeds or kernels, whereas for corn, soybean, and common beans, *T_g_* was measured using seed embryos, axes, and powders. Since water distribution within a seed is not uniform, destructive sample preparation techniques could alter water distribution (Munz et al. 2017), leading to variations in the observed plasticization behavior.

### 4.4 Application and Industry Relevance of the Developed State Diagram

The developed state diagram (Figure 9) provides valuable insights into the water status and material state of canola as MC and temperature change, assisting in the selection of suitable conditions for both drying and storage. This tool holds broad potential for the canola industry. For example, in hot‐air drying, the temperature can be adjusted based on the water state of the canola. Selecting a hot‐air temperature that facilitates the transition from a glass to a rubbery state will increase moisture diffusion, ultimately decreasing drying time (Li et al. 2016). Similarly, state diagrams have historically supported informed decisions in drying rough rice (Ameyaw Owusu et al. 2024; Perdon et al. 2000) and corn (Zheng et al. 2022). For sub‐zero temperature operations (drying and storage), the cold‐air temperature can be strategically selected by considering the thermal transition of both oil and water. This allows for the selection of a critical MC to avoid ice production in canola seeds and the determination of optimum conditions for atmospheric freeze‐drying of wet canola.

It is important to acknowledge that this study has several limitations. DSC, the primary technique used, may not have detected a small amount of freezable water. Furthermore, the determined critical MC was higher than that reported by a recent germination study (Tiwari and Jian 2025). Additionally, this study focused narrowly on storage and drying applications and utilized only one canola variety (LP223). Since different varieties may exhibit different water statuses at the same MC, more comprehensive studies are required to ensure wider industry application and to validate the state diagram across various genetic backgrounds.

## 5 Conclusions

This study used DSC to characterize the unfreezable and freezable water states in canola seeds across a wide range of moisture contents (MCs) (3.4% to 35.8%) and in canola oil. By examining the effects of MC on unfreezable and freezable water content, the study provides insights into the thermal and physical properties of canola seeds under various conditions. The study drew the following conclusions:

1. Freezing and melting enthalpy were affected by the cooling rate, but not by the heating rate.
2. The freezing point of the oil in the seed was about −25°C, and oil in the seed affected the freezing and melting parameters. Therefore, canola seed will be frozen when cooled below −25°C due to oil freezing, irrespective of canola MC.
3. The freezing point of canola seed was about −26°C to −6°C. For canola with <17.8% MCs, the freezing point varied minimally with MC due to oil freezing. Above 17.8% MCs, water influenced the freezing point, and the freezing point increased with the MC.
4. The melting enthalpy (28.57 ‐ 94.05 J/g) increased with MC. For canola <17.8% MCs, melting enthalpy was mainly due to oil melting, and above 17.8% MC, melting enthalpy dramatically increased due to the increased amount of free water.
5. The critical MC was about 16% MC. Below this MC, all the water, including bound, semi‐bound, and free water, exists as unfreezable water. Above this MC, freezable water appears due to an increased amount of free water.
6. Unfreezable water content increased non‐linearly with MC because the water content in seed at <16% MCs would exist as unfreezable water, and freezable water would first appear around 16% MC. Whereas the freezable water content increased linearly with MC.
7. Three glass transitions were observed. However, *T_g1_* was not detected above 6.2% MCs. *T_g2_* (80.79°C–63.37°C) and *T_g3_* (96.65°C–73.38°C) decreased linearly with increasing MC from 3.4% to 35.8%.

## Author Contributions

**Vijay Balaji Kalyanakumar**: investigation, writing ‐ original draft, methodology, validation, visualization, software, formal analysis, data curation. **Fuji Jian**: conceptualization, funding acquisition, methodology, writing ‐ review and editing, software, project administration, supervision, resources. **Trust Beta**: funding acquisition, writing ‐ review and editing, supervision.

## Funding

This research was partially funded by the Canada Foundation for Innovation (CFI, Project number 41866); the Research Manitoba Innovation Proof‐Of‐Concept Grant; and the University of Manitoba Go EC‐Seed project.

## Conflicts of Interest

The authors declare no conflicts of interest.

## Data Availability

Data will be made available on request.
